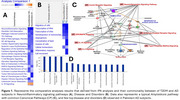# Chronic Type 2 Diabetes and Future Risks of Alzheimer’s Disease in Southeast Asian population

**DOI:** 10.1002/alz70861_108679

**Published:** 2025-12-23

**Authors:** Zarish Noreen, Tanmoy Mondal, Abhay Moghekar, Gail Nunlee‐Blnad, Somiranjan Ghosh Ghosh

**Affiliations:** ^1^ International Islamic University, Islamabad, Federal Pakistan; ^2^ Howard University, Washington, DC USA; ^3^ Johns Hopkins University School of Medicine, Baltimore, MD USA; ^4^ Howard University Hospital, Washington DC, DC USA; ^5^ Howard University, Washington DC, DC USA

## Abstract

**Background:**

Alzheimer’s disease (AD) is a progressive and complex neurodegenerative disorder with a global prevalence that is rapidly increasing, particularly among individuals aged 65 and older. Recent premises have suggested that chronic Type 2 Diabetes (T2DM) poses a higher risk of AD and shares some common pathobiology, which could lead to the development of early diagnostic biomarkers. Although nearly 120 biomarkers are in clinical use, most rely on costly, complex methods that limit access in developing countries. We propose that the development of low‐cost, minimally invasive whole blood transcriptomic markers is urgent and could identify AD at a very early stage, particularly in individuals with chronic T2DM in South Asian populations, especially in Pakistan.

**Method:**

We leveraged our own published data on the Pakistani population to conduct a comparison analysis of molecular networks through gene expression profiling (Transcriptomic) and Ingenuity Pathway Analysis (IPA) using whole‐blood. This additional analysis involved a subgroup of n=18 participants and aimed to identify overlapping gene networks and canonical pathways implicated in both diseases. A TaqMan Low density Array (TLDA) array was used to identify genes associated with Amyloid processing and selected pathways (included genes implicated in multiple secondary steps of beta‐amyloid aggregation, tau hyperphosphorylation, excitotoxicity, inflammation, oxidation and microglial activation).

**Result:**

The results obtained so far have revealed a significant (*p*‐value 0.05) overlap in the dysregulated genes linked to the *Neuroinflammation Signaling Pathway*, *IL‐3 Signaling, TREM1 Signaling; Parkinson’s Signaling Pathway; IL‐10 signaling, PPARα/RXRα Activation* along with *ERK/MAPK Signaling, G‐Protein Coupled Receptor Signaling, and Reelin Signaling in Neurons (Figure 1)*. This indicates shared disruptions in cell signaling and neuronal functions.

**Conclusion:**

These updated analyses and additional findings suggest a shared pathogenic mechanism in T2DM and AD, which could have a profound impact on future research and early disease risk assessment in these populations.